# Predicting synthetic lethal interactions in human cancers using graph regularized self-representative matrix factorization

**DOI:** 10.1186/s12859-019-3197-3

**Published:** 2019-12-24

**Authors:** Jiang Huang, Min Wu, Fan Lu, Le Ou-Yang, Zexuan Zhu

**Affiliations:** 1 College of Computer Science and Software Engineering, Shenzhen University, Nanhai Ave 3688, Shenzhen, 518060 China; 2 Institute for Infocomm Research (I2R), A*STAR, 1 Fusionopolis Way, Singapore, Singapore; 3 Guangdong Key Laboratory of Intelligent Information Processing and Shenzhen Key Laboratory of Media Security, College of Electronics and Information Engineering, Shenzhen University, Nanhai Ave 3688, Shenzhen, 518060 China; 4 Shenzhen Institute of Artificial Intelligence and Robotics for Society, Shenzhen, China

**Keywords:** Synthetic lethality, Graph regularization, Matrix factorization

## Abstract

**Background:**

Synthetic lethality has attracted a lot of attentions in cancer therapeutics due to its utility in identifying new anticancer drug targets. Identifying synthetic lethal (SL) interactions is the key step towards the exploration of synthetic lethality in cancer treatment. However, biological experiments are faced with many challenges when identifying synthetic lethal interactions. Thus, it is necessary to develop computational methods which could serve as useful complements to biological experiments.

**Results:**

In this paper, we propose a novel graph regularized self-representative matrix factorization (GRSMF) algorithm for synthetic lethal interaction prediction. GRSMF first learns the self-representations from the known SL interactions and further integrates the functional similarities among genes derived from Gene Ontology (GO). It can then effectively predict potential SL interactions by leveraging the information provided by known SL interactions and functional annotations of genes. Extensive experiments on the synthetic lethal interaction data downloaded from SynLethDB database demonstrate the superiority of our GRSMF in predicting potential synthetic lethal interactions, compared with other competing methods. Moreover, case studies of novel interactions are conducted in this paper for further evaluating the effectiveness of GRSMF in synthetic lethal interaction prediction.

**Conclusions:**

In this paper, we demonstrate that by adaptively exploiting the self-representation of original SL interaction data, and utilizing functional similarities among genes to enhance the learning of self-representation matrix, our GRSMF could predict potential SL interactions more accurately than other state-of-the-art SL interaction prediction methods.

## Background

Cancers are complex diseases that caused by the defects of multiple genes. Exploring the genetic interactions within cancer cells is important for understanding the mechanisms of cancers. Synthetic lethality, which is a kind of genetic interaction, has attracted a lot of attentions in cancer therapeutics due to its utility in identifying new anticancer drug targets [1, 2]. Synthetic lethality arises between two genes if the combination of deficiencies in the expression of these two genes causes cell death, whereas a deficiency in only one of these two genes will not affect the cell viability [3–6]. Thus, targeting a nonessential gene that has a synthetic lethal (SL) interaction with a tumour-specific mutated gene would be an effective cancer therapy [7], because only tumour cells which harbour this mutation would be killed. In recent years, high-throughput wet-lab screenings such as chemical libraries [8], pooled RNA interference [9, 10] and CRISPR-based genome editing technology [11, 12] have been conducted for searching SL interactions. However, due to the limitations of wet-lab screenings such as high cost, off-target effects and unclear mechanisms [2], efficient computational methods are needed to serve as useful complements for wet-lab screenings.

Recently, various computational algorithms have been proposed to predict SL interactions [13]. According to the principle of the model, existing methods can be roughly classified into three categories: knowledge-based methods, supervised machine learning methods and matrix factorization methods. Knowledge-based methods utilize the knowledge or hypotheses about SL interactions to predict potential SL interactions. For example, based on the assumption that SL interactions tend to take place between genes that are co-expressed, Jerby-Arnon et al. [4] developed a method named DAISY to predict SL interactions from short hairpin RNA (shRNA), somatic copy number alternation (SCNA) and gene expression profiles. Similarly, Sinha et al. [14] proposed a method named Mining Synthetic Lethals (MiSL) to identify mutation-specific SL interactions for specific cancers from pan-cancer human tumour data. However, knowledge-based methods rely heavily on the knowledge of other genomic data, and do not exploit the underlying mechanisms of known SL interactions [2]. Supervised machine learning methods utilize existing SL interactions to build up classification models which could be used to predict novel SL interactions. Based on available SL interactions of yeast, various classification models such as maximum likelihood estimation (MLE) [15], support vector machines (SVM) [15] and ensemble classifiers [16], have been developed for predicting SL interactions. Traditional supervised machine learning methods require both positive and negative training data being available for learning. However, for SL interaction prediction, there are only positive data, and no negative data is available. Matrix factorization methods have become popular for link prediction in recent years due to their utilities in capturing the underlying mechanisms of observed links and incorporating extra relevant information. For instance, Liu et al. [2] formulated SL interaction prediction in human cancer as a logistic matrix factorization problem and assigned higher importance weights for validated SL interaction pairs than unknown pairs. Furthermore, to promote the accuracy of predicted results, they incorporated protein-protein interaction (PPI) similarity and Gene Ontology (GO) similarity into their model. However, the performance of matrix factorization methods depend on the choice of the dimensionality of the latent space which is usually previously unknown and hard to determine.

To address the above problems, in this paper, we introduce a novel graph regularized self-representative matrix factorization (GRSMF) model for SL interaction prediction. Based on known SL interactions, our method focuses on learning a representation matrix from the original input data, which could capture the similarities between genes based on their SL interaction partners. Moreover, GRSMF also draws support from the function similarities among genes that derived from Gene Ontology (GO) annotations to enhance the prediction accuracy. Experiment results on SynLethDB dataset demonstrate that compared with other competing interaction prediction methods, our GRSMF model could achieve more accurate prediction results. Furthermore, case studies of predicting novel SL interactions also demonstrate the effectiveness of GRSMF in predicting SL interactions in human cancer.

## Results

In this section, we demonstrate the performance of GRSMF on SynLethDB database [17]. We perform the sensitivity analysis for parameters in GRSMF to show their impact on prediction performance. Furthermore, we conduct case studies to show the top SL pairs identified by our method.

### Experimental data

We test the performance of GRSMF and compare it with existing methods on SynLethDB database. We also use the GO similarity matrix as graph regularization term in GRSMF.

#### SynLethDB

Currently, SynLethDB is the most comprehensive database for human SL pairs. It collects SL pairs of human and other four model species from four different sources: (1) biochemical experiments, (2) related databases (Syn-lethality [18], Decipher https://decipher.sanger.ac.uk/, GenomeRNAi http://www.genomernai.org/[19], BioGRID https://thebiogrid.org/ [20]), (3) text mining [17] and (4) computational predicted method DAISY [4]. After removing the duplicate human SL pairs, we obtained 19,667 human SL interaction pairs involving 6,375 genes.

#### GO similarity

As demonstrated in [2], functional similarity among genes based on their GO annotations can promote the performance for SL interaction prediction. Therefore, we also utilize the GO similarities among genes for SL interaction prediction in our GRSMF method. We obtain the GO similarity matrix by using the same method presented in [21] and similarly we only consider the biological process (BP) terms in GO.

### Experimental setting

In our experiments, we compare our proposed GRSMF against two existing methods, namely SL^2^MF [2] and BLM-NII [22]. We choose these two methods since they are the latest state-of-the-art SL interaction prediction methods [2]. We also test the performance of GRSMF without the graph regularization and we denote this variant as SMF. These 3 methods are summarized as follows.

- **SL**^**2**^**MF** predicts the SL pairs based on logistic matrix factorization and it trains the model by assigning higher importance weights for known SL pairs than unknown pairs. The parameters *c*, *λ* and *α* are set to 50, 0.01 and 1.0 respectively. In addition, the number of nearest neighbors in GO similarity graph *k*_1_ is set to 150.
- **BLM-NII** was originally designed for drug-target interaction prediction. It was applied for SL prediction in [2] and thus we also implemented it for comparison in this study. In BLM-NII, we set the value of the linear combination weight in the range of {0, 0.1, 0.2, …, 1.0}.
- **SMF** is a variant of GRSMF without the graph regularization. The parameter *λ* in SMF is set to 2^−7^.

For GRSMF, it has two parameters *λ* and *β* and we adopt grid search to select the optimal values for them from the range { 2^−8^,2^−7^,…,2^4^,2^5^}. In particular, *λ* and *β* are set to 2^−7^ and 2^−5^ respectively.

In addition, we adopt 5-fold cross-validation in our experiments for performance evaluation and comparison. The known SL pairs are equally split into 5 non-overlapping subsets. We iteratively use 1 subset for testing and the remaining for training in 5-fold cross validation and use the AUC score (i.e., area under the ROC curve) as our evaluation metric.

### Experimental results based on 5-fold cross validation

**Fig. 1:**
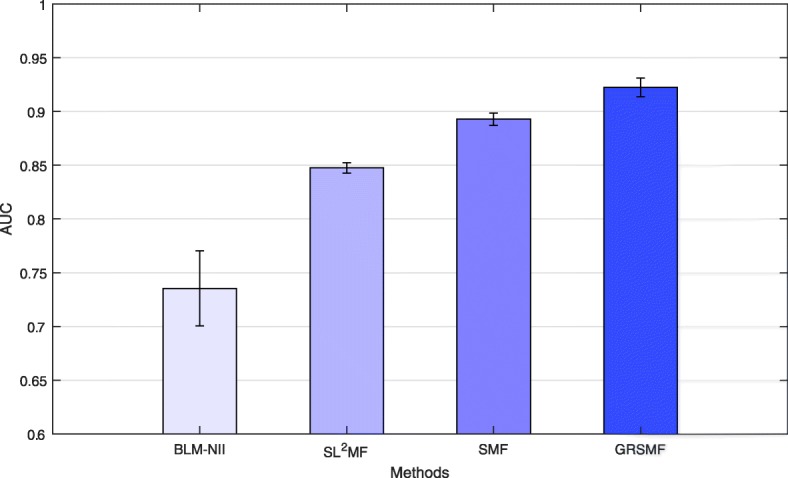
AUC scores of various algorithms in SynLethDB dataset. Error bars denote confidence intervals

Figure 1 shows the performance of various methods on SynLethDB. As shown in Fig. 1, GRSMF achieves an AUC of 0.923 and significantly outperforms BLM-NII (0.735), SL^2^MF (0.847) and SMF (0.893). In particular, we can also have the following two observations based on the comparison in Fig. 1.

First, SMF (i.e., without graph regularization using GO similarity matrix) outperforms SL^2^MF significantly, showing that self-representative matrix factorization is very effective for SL prediction. Unlike SL^2^MF that projects genes into a latent space and predict the SL interaction probability between two genes based on the inner product of their latent vectors, SMF adaptively learns the intra-similarities among genes from the observed SL interactions, according to representation rule introduced in Eq. (1). Thus, SMF has data-adaptiveness and avoids the determination of some sensitive parameters such as the dimension of latent space. Second, graph regularization using GO similarity matrix can boost the prediction performance by 3% from 0.893 (SMF) to 0.923 (GRSMF), demonstrating that functional information of genes is very important for SL interaction prediction. This is also consistent with the results reported in [2]. For these two reasons, GRSMF achieves the highest performance and shows its effectiveness and superiority for SL prediction.

We analyze the running time of various methods. All the experiments are conducted on a laptop with 3.6 GHz CPU and 32G memory. The results are shown in Table 1. We can find from this table that our GRSMF takes the longest running time. However, predicting potential SL interactions based on computational methods is usually used as an offline process, where producing an accurate and reliable prediction result is the primary goal. Therefore, our method is still meaningful.

**Table 1:** The running time of various methods

	GRSMF	BLM-NII	SL^2^MF
Time	1361s	1263s	383s

### Effect of parameters

Recall that GRSMF has two parameters, namely, *λ* and *β* to control the influence of *l*_2_ regularization and graph regularization respectively. In this section, we study the impact of these parameters on GRSMF’s prediction performance.

**Fig. 2:**
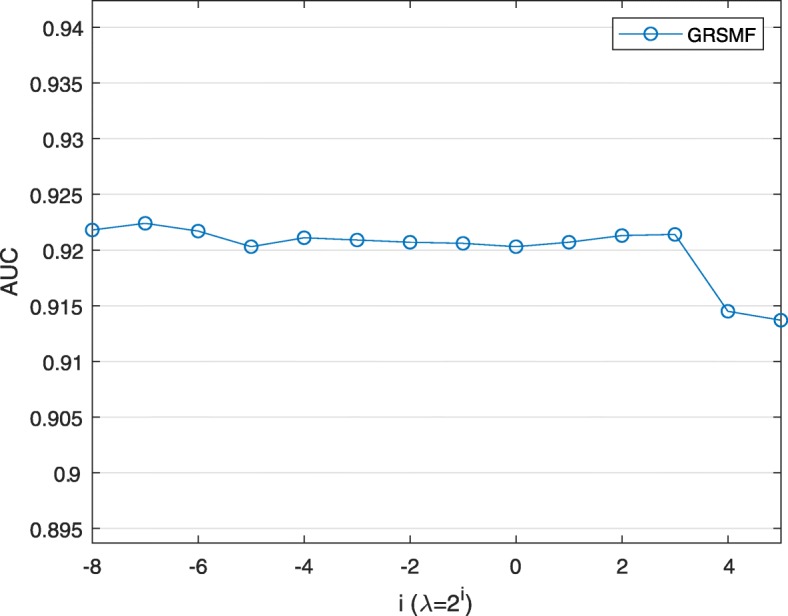
Performance of GRSMF with different values of *λ* while *β* is set to 2 ^−5^

In Fig. 2, we first fix *β* as 2 ^−5^ and set *λ* to different values. We can observe that the AUC scores of GRSMF under different *λ* values are quite close, indicating that our GRSMF model is not very sensitive to the parameter *λ*.

**Fig. 3:**
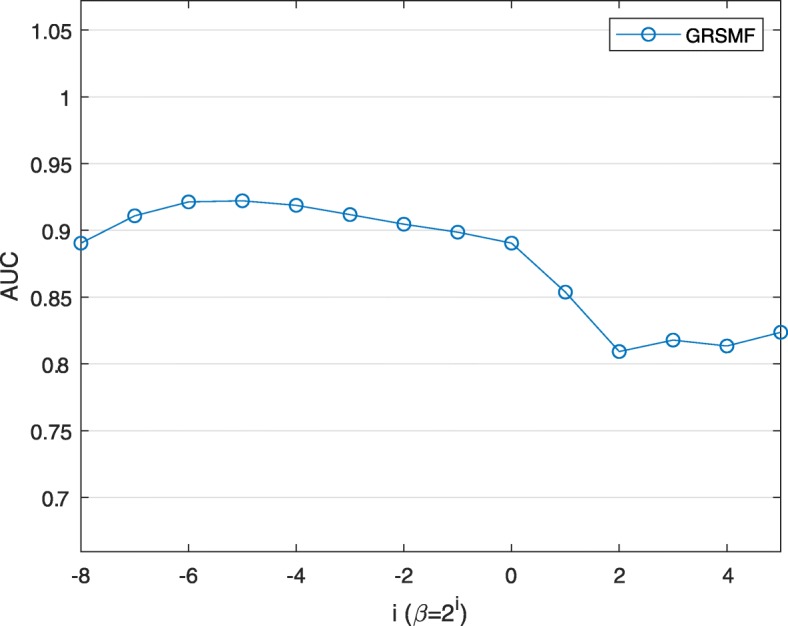
Performance of GRSMF with different values of *β* while *λ* is set as 2 ^−7^

In Fig. 3, we fix *λ* as 2 ^−7^ and then investigate the impact of graph regularization using GO similarity. As shown in Fig. 3, large values of *β* usually lead to poor performance while small values (e.g., 2 ^−6^ and 2 ^−5^) result in very good performance of GRSMF. Based on the results in Fig. 3, our overall conclusion is that SMF in GRSMF plays a critical role for SL interaction prediction, while graph regularization can further help to improve the prediction performance.

### Case studies

We further take all the known SL pairs in SynLethDB as training data and apply GRSMF to predict novel SL pairs. In particular, we rank the unknown pairs in *X* in descending order based on their interaction scores predicted by GRSMF. Thus, the top-ranked pairs are more likely to be potential SL pairs. In Table 2, we show the top-10 SL pairs as well as their scores predicted by GRSMF. Here, the score of each SL pair is obtained from the predicted label marix $\hat {X}$, as described in Algorithm 1.

**Table 2:** The predicted top 10 novel SL gene pairs

Rank	Gene 1	Gene 2	Predicted interaction scores
1	UNG	IGFBP3	0.9242
2	POLD4	MAPK12	0.9129
3	IGHMBP2	CDK4	0.9118
4	XRCC2	MAPK12	0.8605
5	XRCC2	IGHMBP2	0.8603
6	PARP2	PMS2	0.8289
7	PARP2	NHP2	0.8235
8	PARP2	CDK2	0.8151
9	PARP2	IGFBP3	0.8123
10	PARP2	IGHMBP2	0.8049

The gene XRCC2 is involved in the repair of DNA double-strand breaks by homologous recombination. The gene IGHMBP2 also has the functions including DNA binding, DNA recombination, DNA repair and DNA replication. XRCC2 and IGHMBP2 (i.e., 5^*th*^ pair) have back-up functions like DNA repair [23] and thus they have high likelihood to be a SL pair. In addition, MAPK12 is associated with breast cancer, while XRCC2 is part of BCDX2 complex, which acts downstream of BRCA2 recruitment and upstream of RAD51 recruitment [24]. It also makes sense that XRCC2 and MAPK12 (i.e., 4^*th*^ pair) is predicted as a potential SL pair.

It is well-known that poly ADP-ribose polymerases (PARPs) form SL interactions with the two breast cancer genes BRCA1 and BRCA2 [25]. As shown in Table 2, there are 5 predicted SL pairs involving PARP2. In particular, the gene PMS2 interacts with both BRCA1 and BRCA2 [20], and GRSMF predicts that PARP2 and PMS2 have SL interactions (i.e., 6^*th*^ pair in Table 2).

## Discussion

Synthetic lethality is a new angle for cancer therapeutics. Computational methods have been proposed to predict potential SL interactions, which can greatly reduce the costs of biological experiments. In this paper, we present a novel model, named graph regularized self-representative matrix factorization (GRSMF) algorithm, to identify potential SL interactions among genes. Our model focuses on self-representative matrix factorization and also integrates GO similarity matrix as graph regularization. Compared with previous matrix factorization models, we do not need to determine the dimensionality of the latent space and directly learn the similarities among genes based on observed SL interactions. Experiment results on SynLethDB database demonstrate that our GRSMF achieves better performance than other competing methods. Case studies on our predicted novel SL pairs show that our model can effectively identify some candidate SL pairs for further verification.

## Conclusions

In conclusion, revealing the molecular mechanisms underlying cancers is essential to the treatment of cancers and development of new anticancer drugs. Predicting potential SL interactions via computational approaches not only helps to improve our understanding of the mechanisms underlying cancers, but also provides a effective way to aided anticancer therapies. In this study, we provide an efficient model to predict potential SL interactions. In contrast to most existing matrix factorization models, our model avoids the determination of some sensitive hyper-parameters, which makes it easy to implement. Our model provides a promising strategy to predict potential SL interactions for further experimental verification, and contributes to the treatment of cancers. In the future, we plan to integrate biological knowledge such as pathways, protein domains, TCGA data, etc., to further improve our GRSMF model.

## Methods

In this section, we first describe the notations and formulate the problem, then we propose a novel graph regularized self-representative matrix factorization model and introduce a relaxed Majorization-Minimization algorithm to solve the optimization problem.

### Notations and problem statement

In this paper, a set of genes is denoted by $G=\{g_{i}\}_{i=1}^{n}$, where *n* is the number of genes. A binary association matrix $X = [X_{ij}] \in {0, 1}^{n\times n}$ is used to describe the SL interactions among genes in *G*. If there exists a validated SL interaction between genes *g*_*i*_ and *g*_*j*_, *X*_*ij*_ is set to 1; otherwise, *X*_*ij*_ is set to 0. Here, gene pair (*g*_*i*_,*g*_*j*_) with *X*_*ij*_=0 is referred to as “unknown pair”, since there is no clear evidence to demonstrate whether there is an SL interaction between genes *g*_*i*_ and *g*_*j*_ or not. Note that gene pairs (*g*_*i*_,*g*_*j*_) and (*g*_*j*_,*g*_*i*_) are treated as the same pair, and we set *X*_*ii*_=0 for *i*=1,…,*n*. Therefore, *X* is a symmetric matrix with *X*_*ij*_=*X*_*ji*_.

Given a set of observed SL interactions, the problem of SL interaction prediction is to identify a set of gene pairs that are most likely to have SL interaction from the “unknown pairs”. This task can be achieved by ranking the candidate gene pairs according to the predicted SL propensities in descending orders, and selecting the top-ranked gene pairs as potential SL interactions. In this study, we propose a regularized self-representative matrix factorization model to identify the SL interactions among genes in *G*. Furthermore, based on the functional similarities between genes, we also incorporate a neighborhood regularization in our model to enhance the accuracy of prediction.

### Self-representative matrix factorization model

The proposed model is developed based on self-representative matrix factorization, which has been successfully used in subspace clustering [26]. In traditional self-representative matrix factorization models, the objective is to exploit the representation of the original data *X* in which the data itself is treated as a dictionary, i.e., the input data *X* is self-represented by a linear combination of its columns as *X*≈*XU*, where *U*∈*R*^*n*×*n*^ denotes the coefficient matrix which is a meaningful representation of the columns of *X*. In this study, since the input data *X* is a symmetric matrix, the representations of its rows should be same as the representations of its columns. Thus, we propose a new model in which *X* is self-represented by linear combinations of its rows and columns as *X*≈*U*^*T*^*XU*. For the *i*-th gene *g*_*i*_, *U*_*li*_ can be used to denote the probability of gene *g*_*i*_ being represented by gene *g*_*l*_, which captures the similarity between genes *g*_*i*_ and *g*_*l*_ based on their SL interactions with other genes. To guarantee the probability property of *U*_·*i*_ (which denotes the *i*-th column of *U*), we introduce constraints 0≤*U*_*li*_≤1 and $\sum _{l=1}^{n} U_{li} =1$ for *i*=1,…,*n*. To avoid the trivial solution that only one element in *U*_·*i*_ has value 1 while all the other elements being zeros, we also impose a constraint on *U*_·*i*_. In particular, we choose the *l*_2_ norm $\|U_{\cdot i}\|_{2}^{2} = \sum _{l=1}^{n} U_{li}^{2}$ due to its simplicity and effectiveness.

Hence, by taking into account the above constraints, we have the following regularized self-representative matrix factorization model

1 $$ \begin{aligned} &\min_{U} \|X - U^{T} X U\|^{2}_{F} + \lambda\|U\|^{2}_{F} \\ &s.t. \quad 0 \leq U \leq 1, \quad \sum\limits_{l=1}^{n} U_{li} =1, \quad \text{for} \quad i = 1, \ldots, n. \end{aligned}  $$

where ∥·∥_*F*_ is the Frobenius norm and *λ* is a tuning parameter which controls the influence of the *l*_2_ regularization.

### Graph regularization

In the above objective function (1), the representation matrix *U* is learned from the original data matrix *X*, which makes it sensitive to the input data *X*. If the input data only covers very few known SL interactions (which means most of the elements in *X* are zeros), it may be hard to learn a comprehensive representation matrix. Therefore, we would like to incorporate some prior information that can reflect the similarities among genes into our model. In particular, based on the assumption that genes with similar functions tend to have similar presentations, we take into account the Gene Ontology (GO) semantic similarities among genes to promote the determination of representation matrix and improve the prediction performance. Let *S*∈*R*^*n*×*n*^ denote the GO similarity matrix where *S*_*ij*_ describes the functional similarity between genes *g*_*i*_ and *g*_*j*_. The value of *S*_*ij*_ is ranging from 0 to 1, where the larger the value of *S*_*ij*_, the more similar the corresponding two genes. In this study, we adopt the method presented in [21] to calculate the Gene Ontology (GO) semantic similarities among genes. The graph regularization based on *S* is defined as follows

2 $$ R = \frac{1}{2} \sum_{l = 1}^{n} \sum_{i, j = 1}^{n} \|U_{il} - U_{jl}\|^{2} S_{ij} = Tr(U^{T} L U).  $$

where *Tr*(·) denotes the trace of a matrix, *D* is a diagonal matrix with $D_{ii} = \sum _{j=1}^{n} S_{ij}$ and *L*=*D*−*S*.

### Graph regularized self-representative matrix factorization model

By incorporating the above graph regularization term (2) into Eq. (1), the final objective optimization function of our graph regularized self-representative matrix factorization (GRSMF) model is formulated as follows.

3 $$ \begin{aligned} &\min_{U} \|X - U^{T} X U\|^{2}_{F} + \lambda\|U\|^{2}_{F} +\beta {\rm{Tr}}(U^{T} L U),\\ &s.t. \quad 0 \leq U \leq 1, \quad \sum\limits_{l=1}^{n} U_{li} =1, \quad \text{for} \quad i = 1, \ldots, n. \end{aligned}  $$

where the parameter *β* controls the effect of graph regularization.

### Optimization algorithm

In this study, we solve the objective function (3) based on relaxed Majorization-Minimization [27] method. Particularly, the objective function in Eq. (3) is denoted as $\mathcal {J}$ and ▽_*U*_ denotes the gradient of our objective function with respect to *U*.

4 $$ {{}\begin{aligned} &\bigtriangledown_{U}\,=\,4(X U)\left(U^{T} X U\right) \,+\, 2 \lambda U \,+\, 2 \beta (D U) \,-\, 4(X U) X \,-\,\! 2 \beta S U. \end{aligned}}  $$

Let $\bigtriangledown ^{+}_{U} = 4(XU)(U^{T} X U) + 2 \lambda U+2 \beta (D U)$ and $\bigtriangledown ^{-}_{U}=4(XU) X + 2 \beta S U$ denote the positive and negative parts of ▽_*U*_ respectively. Then we have $\bigtriangledown _{U} =\bigtriangledown _{U}^{+} - \bigtriangledown _{U}^{-}$.

Due to the constraint $\sum \limits _{l=1}^{n} U_{li} =1$ and 0≤*U*_*li*_≤1, we obtain the following updating rule for *U*_*li*_:

5 $$ U_{li} =U_{li} \cdot \dfrac {a_{i}{(\bigtriangledown^{-}_{U})}_{li}+1}{a_{i}{(\bigtriangledown^{+}_{U})_{li}+b_{i}}}.  $$

where *a*_*i*_ and *b*_*i*_ can be obtained by Eqs. 6 and 7 respectively.

6 $$ a_{i} =\sum_{l = 1}^{n} \frac{U_{li}} {(\bigtriangledown^{+}_{U})_{li}},  $$

7 $$ b_{i}=\sum_{l = 1}^{n} U_{li}\frac{(\bigtriangledown^{-}_{U})_{li}}{(\bigtriangledown^{+}_{U})_{li}}.  $$

The details of the optimization algorithm are described in Algorithm 1. *U* can be updated by Eq. (5). In this study, we stop the iteration until the relative change of objective function $\mathcal {J}$ is less than 1*e*−4, i.e., $\frac {\|\mathcal {J}^{(t+1)} - \mathcal {J}^{(t)}\|_{1}}{\|\mathcal {J}^{(t)}\|_{1}} < 1e-4$, where $\mathcal {J}^{(t)}$ denotes the value of objective function at *t*-th iteration. Finally, the predicted label matrix $\hat {X}$ can be computed by ${\hat {X} = U^{T} X U}$ when the algorithm arrives at the convergence conditions.


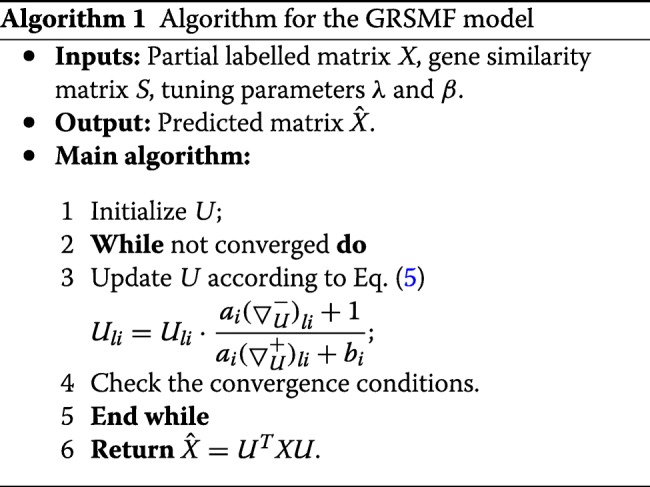


## Data Availability

All the experimental results and code can be downloaded from https://github.com/Oyl-CityU/GRSMF.

## Abbreviations

AUC: Area under the curve
BLM-NII: Bipartite local model with neighbor-based interaction-profile inferring
BP: Biological process
GO: Gene ontology
GRSMF: Graph regularized self-representative matrix factorization
MLE: Maximum likelihood estimation
PPI: Protein-protein interaction
ROC: Receiver operating characteristic
SL: Synthetic lethal
SMF: Self-representative matrix factorization
